# The use of venoarterial ECMO as a successful strategy in acute severe mitral regurgitation secondary to papillary muscle rupture due to acute myocardial infarction: A case report and narrative review

**DOI:** 10.1097/MD.0000000000044646

**Published:** 2025-09-19

**Authors:** Yan Wang, Jie Wang, Caihong Sun, Tao He, Weijie Wang, Wenhui Zhang, Yan Wang, Zehui Qin, Leibing Li

**Affiliations:** a Medical-Surgical Intensive Care Unit, Rizhao Clinical Research Center for Acute and Critical Care, People’s Hospital of Rizhao, Jining Medical University, Rizhao, Shandong, China; b Department of General Practice, People’s Hospital of Rizhao, Jining Medical University, Rizhao, Shandong, China.

**Keywords:** cardiogenic shock, case report, extracorporeal membrane oxygenation, mechanical circulatory support, myocardial infarction

## Abstract

**Rationale:**

Acute mitral regurgitation (MR) secondary to papillary muscle rupture is a rare but often life-threatening mechanical complication post-acute myocardial infarction (MI). The use of peripheral venoarterial extracorporeal membrane oxygenation (VA-ECMO) as a bridge to mitral valve replacement surgery may improve outcome of such patients.

**Patient concerns:**

We reported the case of a 70-year old woman with past history who presented to the emergency department at People’s Hospital of Rizhao with “a 5-day history of chest distress.” She developed refractory cardiogenic shock, severe pulmonary edema and severe acidosis.

**Diagnoses:**

Restoration of spontaneous circulation following PCI, VA-ECMO, IABP, and early mitral valve replacement.

**Interventions:**

After performing percutaneous coronary intervention (PCI) supported by VA-ECMO and intra-aortic balloon pump (IABP), our group performed a early mitral valve replacement for this patient.

**Outcomes:**

This patient preliminarily made a good recovery after VA-ECMO and IABP discontinued.

**Lessons:**

This case demonstrated that VA ECMO combined with PCI, VA-ECMO, IABP and early mitral valve replacement can result in favorable outcomes, and might be viable emergency therapeutic options.

## 1. Introduction

Acute mitral regurgitation (MR) secondary to papillary muscle rupture (PMR) is a rare but often life-threatening mechanical complication post-acute myocardial infarction (MI). In addition, ventricular septal rupture and cardiac free-wall rupture, these complications collectively account for 0.5% to 2% of acute MI cases in contemporary clinical registries.^[1–6]^ These conditions precipitate refractory cardiogenic shock (CS) in up to 60% of patients, with mortality rates exceeding 80% without prompt surgical intervention.^[1,5]^ PMR causes severe MR and result in acute pulmonary edema, hypoxia and refractory CS in the SHOCK trial registry.^[7]^ Survival of medically treated patients is extremely poor, the PMR requires emergent surgical intervention including mitral valve replacement (MVR) and mitral valve repair (MVr) and have a poor long-term prognosis even after surgical correction.^[8,9]^ The current ESC/EACTS myocardial revascularization guidelines assign extracorporeal life support (ECLS) a Class IIb recommendation (Level of Evidence C) for post-AMI mechanical complications.^[10]^ Despite this guidance, clinical experiences specifically investigating venoarterial (VA)-ECLS in AMI-related (PMR) remain sparsely documented. This case shows that the use of ECMO combined with intra-aortic balloon pump (IABP) as a stabilization strategy and a bridge to recovery may potentially improve the outcome of MR post-acute MI.

## 2. Case presentation

**Figure 1.**
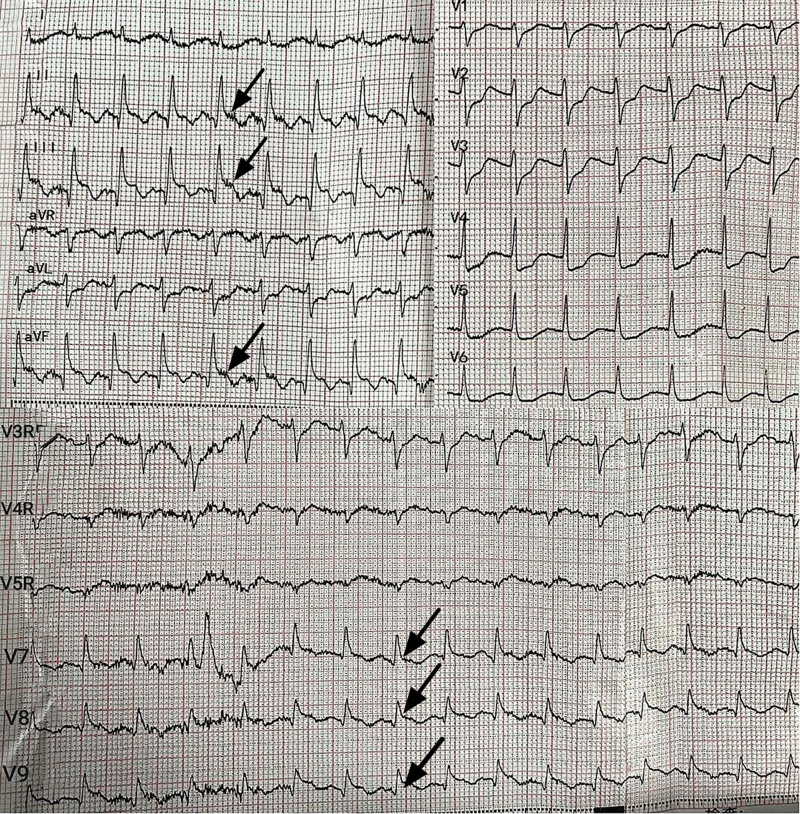
An 18-lead ECG demonstrated ST-T wave changes in leads II, III, aVF, and V7–V8 suspicious (black arrow) for an acute inferior–posterior wall STEMI.

Herein, we reported a case of acute MR that presented with refractory CS, severe MR hypoxemia that required VA-ECMO combined with emergent percutaneous coronary intervention (PCI) and MVR. A 70-year-old previously healthy woman (with hypertriglyceridemia but no history of cardiac disease) was admitted to People’s Hospital of Rizhao on September 27, 2024, with “a 5-day history of chest distress.” The vital signs and physical examination of patient at admission showed: a body temperature of 36.6°C, a heart rate of 88 times/min, respiratory rate 21 times/min, and blood pressure of 95/68 mm Hg. The points of Glasgow Coma Scale score was 15 (E4V5M6). The heart sounds was low and dull, and no murmur in each valve area. Other positive clinical sign was not found. Routine admission laboratory tests showed: cardiac troponin I (TnI) 1.525 ng/mL, creatine kinase-MB (CK-MB) 6.07ng/mL, NTpro B-type natriuretic peptide (NTpro-BNP) 1669.3 pg/mL. Electrocardiogram (ECG) revealed that acute inferior ST-elevation MI. Transthoracic echocardiography (TTE) revealed that segmental movement abnormalities in the posterior inferior wall of the left ventricle accompanied mild ventricular hypokinesia (left ventricular ejection fraction (LVEF) 53%). She was suspected to have acute MI, antiplatelet (Clopidogrel Bisulfate tablets and Indobufen tablets) and lipid regulation (Rosuvastatin Calcium Tablets) treatments were given. Five hours later, the patient presented with sudden-onset chest tightness, dyspnea, profuse diaphoresis, and restlessness. Physical examination revealed hemodynamic instability: blood pressure 76/58 mm Hg, heart rate 115 bpm, and oxygen saturation (SpO_2_) 92% (FiO2 50%). Pulmonary auscultation demonstrated coarse breath sounds with extensive bilateral crackles, while cardiac examination identified a regular rhythm with a grade 2/6 systolic murmur at the mitral auscultation area. Despite therapeutic interventions including titrated oxygen supplementation, dopamine infusion (8% to 15 μg/kg/min), intravenous furosemide (40 mg bolus), and morphine (5 mg incremental doses), the patient exhibited progressive clinical deterioration characterized by declining oxygen saturation, refractory hypotension, and emergence of copious pink frothy sputum indicative of acute pulmonary edema. So the patient was transferred to intensive care unit immediately because respiratory and circulatory deteriorated rapidly. The vital signs are as follows: tachycardia (144 beats/min), tachypnea (36 breaths/min), hypotension (83/64 mm Hg) and noninvasive oxygen saturation (SPO2) (82%) (FiO2 60%). Wet rales were heard during auscultation in both lung. Heart auscultation disclosed 2/6 systolic murmur in the mitral auscultation area. Arterial blood gas analysis showed that severe pulmonary edema and acidosis (pH 7.3, PaCO2 24 mm Hg, PaO2 62 mm Hg, HCO3 11.8 mmol/L and lactic acid (Lac) 9.5mmol/L). An 18-lead ECG showed ST-segment elevation in leads II, III, aVF, and V7–V9 (Fig. 1). TTE showed that severe acute MR secondary to anterolateral PMR, pulmonary hypertension (45 mm Hg), and an LVEF of 31%.

**Figure 2.**
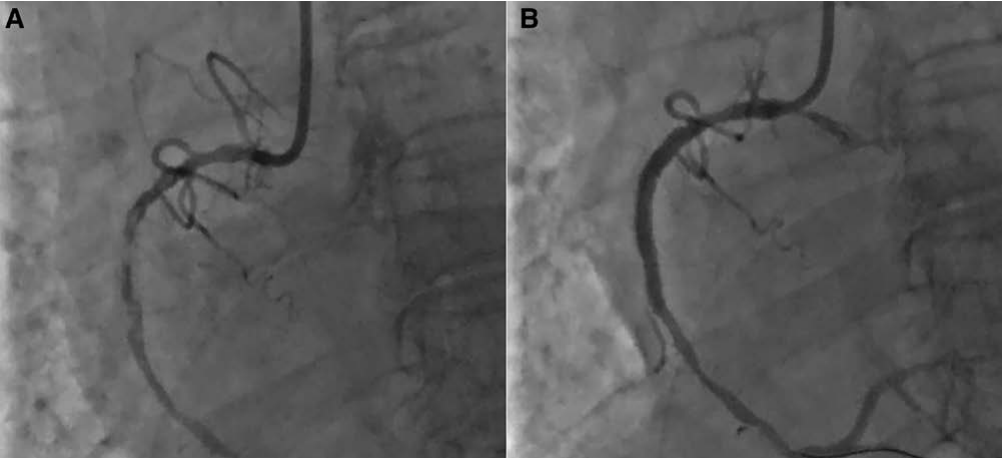
Coronary angiogram revealed occluded right coronary artery (A) and opened right coronary artery (B).

**Figure 3.**
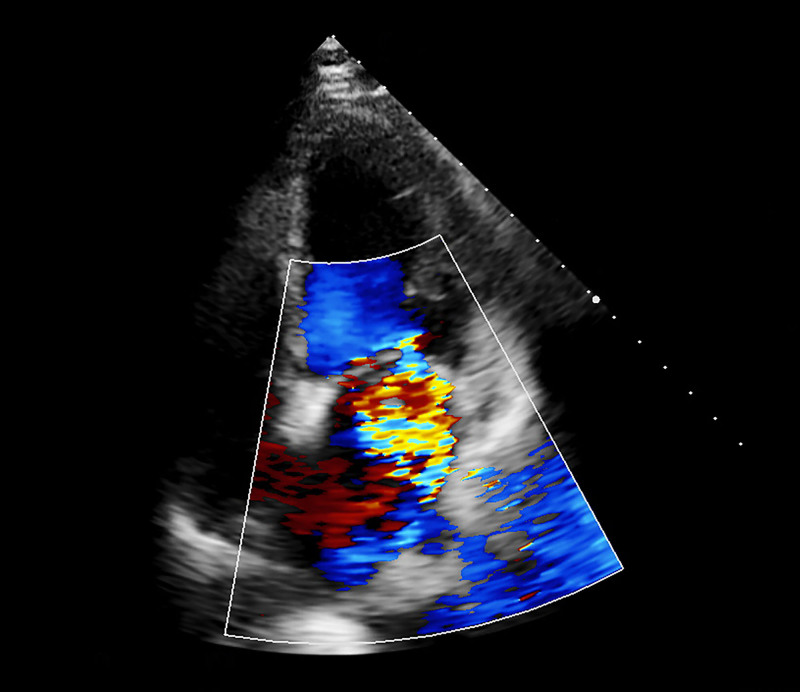
Transesophageal echocardiography still image demonstrating severe mitral regurgitation.

**Figure 4.**
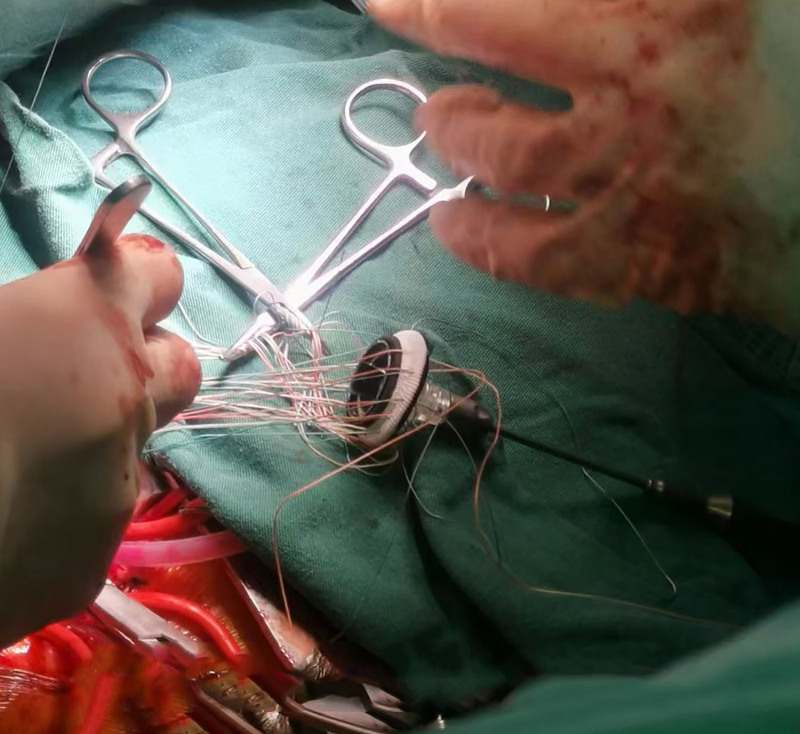
The anterior leaflet of the mitral valve was replaced with size 25 carbomedics mechanical valve.

**Figure 5.**
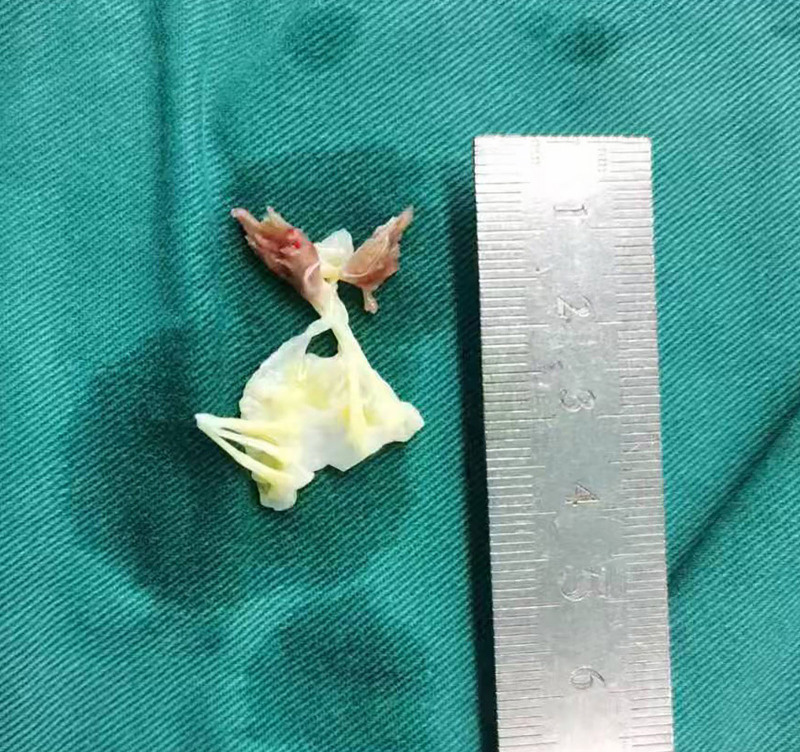
Surgical specimen of anterior mitral leafet.

**Figure 6.**
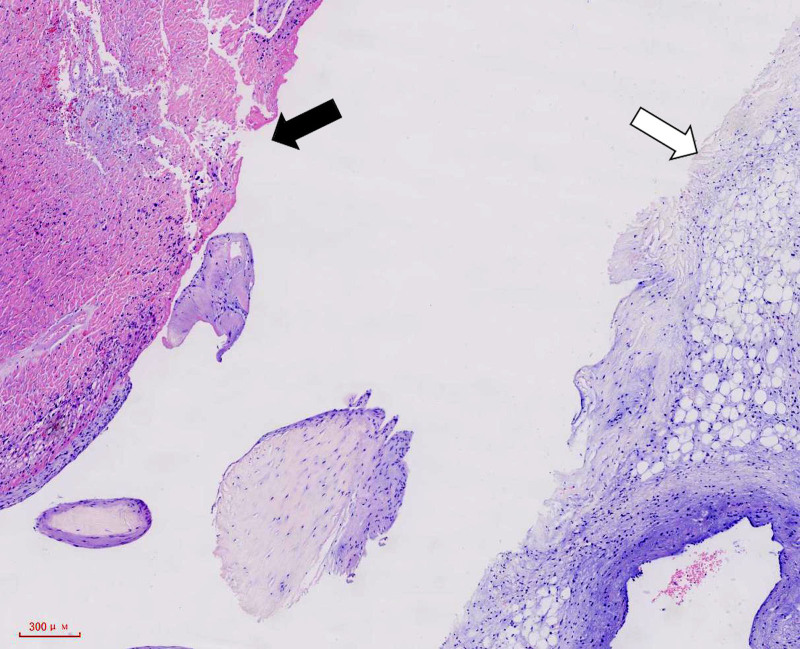
Pathological examination (HE stain) of papillary muscle (black arrow) and mitral valve (white arrow) showing coagulation necrosis of myocytes and mitral valve.

Invasive mechanical ventilation and circulating agonists (norepinephrine 1.5μg/kg/min) were given promptly. However, the vasopressor failed to maintain BP (87/52 mm Hg). Emergency bedside TTE revealed severe prolapse of the anterior mitral leaflet in the A2 segment, accompanied by torrential MR. Additional findings included left ventricular dilatation and markedly reduced systolic function, with LVEF quantified at 23%. VA-ECMO was performed immediately by our ECMO team. The initial settings of VA-ECMO were: 3500 rpm, 80% and 5 L/min of oxygen. Heparin dosing was dynamically adjusted to achieve protocol-defined coagulation targets: activated partial thromboplastin time 60 to 80 seconds or activated coagulation time 180 to 200 seconds. Targeted packed red blood cell transfusions were implemented to maintain hemoglobin > 90 g/L during critical care management, adhering to restrictive transfusion thresholds for hemodynamically unstable patients. This patient subsequently underwent a successful PCI (A 3.5 × 28 mm Nuoyan drug-eluting stent was successfully implanted) on an acutely occluded right coronary artery (Fig. 2). Following PCI, hemodynamic reassessment was conducted after 3 hours of VA-ECMO support(blood flow 3.5L/min,70% and 3 L/min of oxygen). Repeat TTE demonstrated that persistent left ventricular dilatation (end-diastolic diameter 52 mm) with sustained systolic dysfunction (LVEF 28%; fractional shortening 14%). In alignment with contemporary CS management protocols, an IABP (1:1) was inserted for patient after evaluation of ECMO team in order to improve antegrade blood flow and avoid distension and thrombogenesis of left ventricular (Fig. 2). Despite 72 hours of mechanical circulatory support coupled with aggressive volume management via continuous renal replacement therapy (CRRT) and high positive end-expiratory pressure (PEEP 12 cmH2O) mechanical ventilation, the patient exhibited persistent copious pink frothy sputum accompanied by elevated central venous pressure (CVP 18 mm Hg). TEE revealed multiple left ventricular wall motion abnormalities, anterior mitral leaflet prolapse involving A2 and partial A3 segments (Carpentier classification), torrential MR (vena contracta width of 11 mm, regurgitant orifice area of 9.3 cm², and regurgitant fraction of 63%), and pulmonary hypertension (estimated systolic pulmonary artery pressure 44 mm Hg) (Fig. 3). Cardiac Surgery Team performed the MVR for this patient on the third day of admission. The anterior leaflet of the mitral valve was replaced with a size 25 carbomedics mechanical valve while keeping the posterior leaflet intact (Fig. 4). Surgical specimen of anterior mitral leafet is found in Figure 5. Figure 6 shows pathological examination of surgical specimens. Under ECMO support, the internal mammary artery demonstrated non-palpable pulsation with severe tissue edema and hemorrhagic complications encountered during dissection, rendering it nonviable for utilization. Bilateral great saphenous veins exhibited marked varicose dilation that precluded their surgical application. Given these intraoperative constraints and the severe myocardial edema, the coronary artery bypass grafting (CABG) procedure was ultimately aborted.

**Figure 7.**
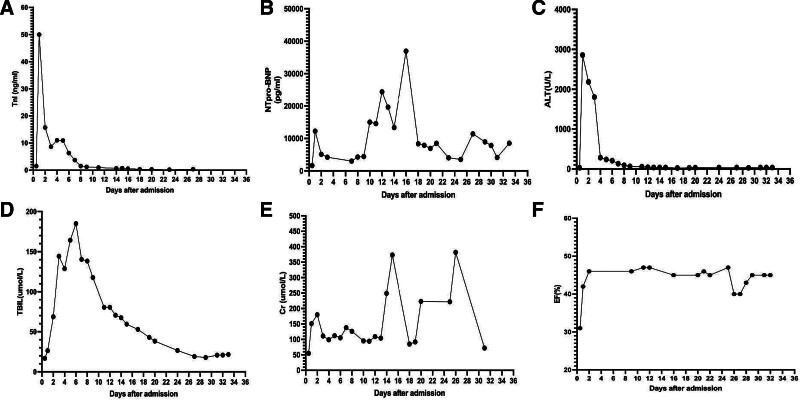
Clinical course of the patient during hospitalization in ICU. Changes in cTnI (A), NT-proBNP (B), ALT (C), TBIL (D), Creatinine (E), and LVEF (F) during hospitalization in ICU.

**Figure 8.**
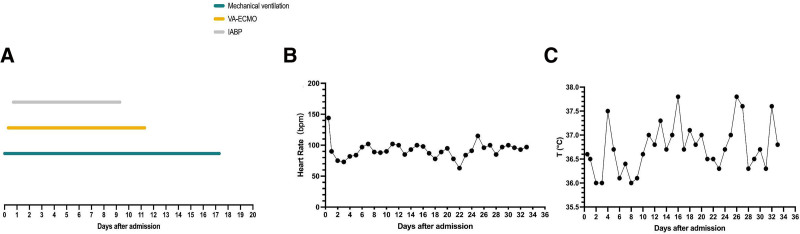
Combined treatment of VA-ECMO, IABP with Mechanical ventilation (A), heart rate (B), and body temperature changes (C).

The subsequent days of treatment were maintained using a combination of ECMO, IABP, CRRT, mechanical ventilation and drug support. After the tenth day of admission, the patient regained consciousness with satisfactory cardiopulmonary compensation: Under low-intensity ventilatory support, the PaO2/FiO2 ratio exceeded 300 mm Hg. With ECMO blood flow titrated to 1.5 L/min, hemodynamic stability was maintained without vasoactive agent requirement. Point-of-care echocardiography demonstrated preserved biventricular function (Velocity-Time Integral 17 cm, LVEF 47%) with absence of left ventricular dilation and intact right ventricular performance. At the same time, the patient’s hemodynamics was stable, and cardiac injury markers such as cTnI and NT-proBNP tended to normal. Consequently, following successful completion of the spontaneous circulation weaning trial under comprehensive hemodynamic monitoring, IABP and ECMO support were systematically discontinued in accordance with institutional weaning protocols.Within 18 days, the patient’s respiratory, circulatory functions, and LV function progressively improved, and the ECMO, endotracheal tube, CRRT, and IABP were gradually removed. The clinical course, vital signs, and associated laboratory tests and examinations are showed during hospitalization (Figs. 7 and 8).

This patient preliminarily made a good recovery after VA-ECMO and IABP discontinued. Written informed consent was obtained from the patient for publication of this case report.

## 3. Discussion

Approximately 6% of acute MI occurs CS.^[11]^ It is reported that the prevalence of PMR following ST-elevation MI is 0.05% to 0.26% of patients.^[12]^ PMR occurs most frequently within the first 7 days after acute MI and the reported median time to PMR is approximately 13 hours.^[1]^ TTE often the initial diagnostic tool to identify the PMR with a sensitivity of 65% to 85% while TEE offers a sensitivity of 95% to 100%.^[13]^ PMR causes severe MR and result in acute pulmonary edema, hypoxia and refractory CS in the SHOCK trial registry.^[7]^

PCI is considered as a class I treatment for acute MI, which also can reduce the necessity of concomitant CABG.^[4]^ To maintain hemodynamics and improve end organ perfusion for severe pulmonary congestion and refractory CS usually need MCS support, such as IABP or ECMO. However, less publications reported the use of MCS for CS with mechanical complications. The use of MCS was associated with a high mortality for acute MI.^[14–16]^ MCS has been described as a bridge to surgery for acute CS. ECMO, IABP, and PCI were given by our group at primary time for this patient with CS, severe MR, and acute pulmonary edema.

In the American College of Cardiology Foundation/American Heart Association and the European Society of Cardiology guidelines, early surgical intervention (repair or replace the valve) is recommended for patients with PMR after acute MI.^[17,18]^ However, repairing weak and friable tissue is challenged, thus, the MVR is the best choice. Mortality rates with early surgical intervention is reported at between 20% and 40% and the mortality without surgery remains high and reaches 80%.^[19]^ It is reported that acute surgical intervention can largely reduce the mortality rate of patient with PMR after MI from 80% to 39%.^[20]^ MVR was performed by our group next day under the VA-ECMO and IABP support. One article^[21]^ showed that a case of acute MR with CS and severe hypoxia followed the insertion of veno-venoarterial (VVA) ECMO and emergency MVR achieved good recovery. However, we performed a VA-ECMO, but not VVA-ECMO because the oxygen saturation was a satisfactory followed performing IABP and adjusting the ventilator parameters. There is no significant difference in early mortality between patients with concomitant CABG and without concomitant CABG.^[6,7]^ However, patients with concomitant CABG can significantly improved long-term survival compared to these without concomitant CABG.^[19]^ Our group just performed a MVR surgery for this patient. However, a CABG was not performed due to severe the myocardial edema and coagulopathy.

## 4. Conclusions

The use of peripheral VA-ECMO combined with IABP as a bridge to MVR surgery. Early diagnosis, medical therapy, MCS support, PCI and MVR surgery are essential for patient with refractory CS, severe MR and acute pulmonary edema. In all, initial management should stabilize the patient’s vital signs and ensure end organ perfusion, with surgery planned timely in the same hospitalization.

## Acknowledgments

The authors thank all the reviewers for their assistance and support and authors of the included studies.

## Author contributions

**Conceptualization:** Caihong Sun, Tao He, Leibing Li.

**Data curation:** Tao He.

**Investigation:** Tao He, Weijie Wang, Leibing Li.

**Methodology:** Caihong Sun, Tao He, Leibing Li.

**Project administration:** Caihong Sun.

**Resources:** Caihong Sun, Leibing Li.

**Software:** Tao He.

**Validation:** Caihong Sun, Tao He, Weijie Wang, Leibing Li.

**Visualization:** Caihong Sun.

**Writing – original draft:** Yan Wang (the younger one), Jie Wang.

**Writing – review & editing:** Yan Wang (the older one), Jie Wang, Caihong Sun, Tao He, Wenhui Zhang, Zehui Qin.

## References

[1] Hochman JS Buller CE Sleeper LA. Cardiogenic shock complicating acute myocardial infarction--etiologies, management and outcome: a report from the SHOCK trial registry. Should we emergently revascularize occluded coronaries for cardiogenic shock. J Am Coll Cardiol. 2000;36:1063–70. 10985706 10.1016/s0735-1097(00)00879-2

[2] Alajaji WA Akl EA Farha A Jaber WA AlJaroudi WA. Surgical versus medical management of patients with acute ischemic mitral regurgitation: a systematic review. BMC Res Notes. 2015;8:712. 26602753 10.1186/s13104-015-1704-9 PMC4659221

[3] Bhardwaj B Sidhu G Balla S. Outcomes and hospital utilization in patients with papillary muscle rupture associated with acute myocardial infarction. Am J Cardiol. 2020;125:1020–5. 31973809 10.1016/j.amjcard.2019.12.051

[4] French JK Hellkamp AS Armstrong PW. Mechanical complications after percutaneous coronary intervention in ST-elevation myocardial infarction (from APEX-AMI). Am J Cardiol. 2010;105:59–63. 20102891 10.1016/j.amjcard.2009.08.653

[5] Russo A Suri RM Grigioni F. Clinical outcome after surgical correction of mitral regurgitation due to papillary muscle rupture. Circulation. 2008;118:1528–34. 18809799 10.1161/CIRCULATIONAHA.107.747949

[6] Kutty RS Jones N Moorjani N. Mechanical complications of acute myocardial infarction. Cardiol Clin. 2013;31:519–31, vii. 24188218 10.1016/j.ccl.2013.07.004

[7] Thompson CR Buller CE Sleeper LA. Cardiogenic shock due to acute severe mitral regurgitation complicating acute myocardial infarction: a report from the SHOCK trial registry. Should we use emergently revascularize occluded coronaries in cardiogenic shock. J Am Coll Cardiol. 2000;36:1104–9. 10985712 10.1016/s0735-1097(00)00846-9

[8] Abu Saleh WK Aljabbari O Ramlawi B Ramchandani M. Case report: necrosis of the anterolateral papillary muscle—an unusual mechanical complication of myocardial infarction. Methodist Debakey Cardiovasc J. 2015;11:48–50. 25793030 10.14797/mdcj-11-1-48 PMC4362066

[9] Hennessey B Sabatovicz N Jr Del Trigo M. Acute ischaemic mitral valve regurgitation. J Clin Med. 2022;11:5526. 36233410 10.3390/jcm11195526 PMC9571705

[10] Sousa-Uva M Neumann FJ Ahlsson A; ESC Scientific Document Group. 2018 ESC/EACTS guidelines on myocardial revascularization. Eur J Cardiothorac Surg. 2019;55:4–90. 30165632 10.1093/ejcts/ezy289

[11] Shah S Ivan E Michaels AD. Cardiogenic shock in acute coronary syndromes. In: Chatterjee K, ed. Cardiology: An Illustrated Textbook. Jaypee Brothers Medical Pub; 2020.

[12] Massimi G Matteucci M De Bonis M. Extracorporeal life support in mitral papillary muscle rupture: outcome of multicenter study. Artif Organs. 2023;47:1386–94. 37039965 10.1111/aor.14541

[13] Sochowski RA Chan KL Ascah KJ Bedard P. Comparison of accuracy of transesophageal versus transthoracic echocardiography for the detection of mitral valve prolapse with ruptured chordae tendineae (flail mitral leaflet). Am J Cardiol. 1991;67:1251–5. 2035450 10.1016/0002-9149(91)90936-f

[14] Elbadawi A Elgendy IY Mahmoud K. Temporal trends and outcomes of mechanical complications in patients with acute myocardial infarction. JACC Cardiovasc Interv. 2019;12:1825–36. 31537282 10.1016/j.jcin.2019.04.039

[15] Formica F Mariani S Singh G. Postinfarction left ventricular free wall rupture: a 17-year single-centre experience. Eur J Cardiothorac Surg. 2018;53:150–6. 28977576 10.1093/ejcts/ezx271

[16] Arnaoutakis GJ Zhao Y George TJ Sciortino CM McCarthy PM Conte JV. Surgical repair of ventricular septal defect after myocardial infarction: outcomes from the Society of Thoracic Surgeons National Database. Ann Thorac Surg. 2012;94:436–43; discussion 443. 22626761 10.1016/j.athoracsur.2012.04.020 PMC3608099

[17] Ibanez B James S Agewall S.; ESC Scientific Document Group. 2017 ESC Guidelines for the management of acute myocardial infarction in patients presenting with ST-segment elevation: the task force for the management of acute myocardial infarction in patients presenting with ST-segment elevation of the European Society of Cardiology (ESC). Eur Heart J. 2018;39:119–77. 28886621 10.1093/eurheartj/ehx393

[18] O’Gara PT Kushner FG Ascheim DD. 2013 ACCF/AHA guideline for the management of ST-elevation myocardial infarction: a report of the American College of Cardiology Foundation/American Heart Association Task Force on Practice Guidelines. J Am Coll Cardiol. 2013;61:e78–e140. 23256914 10.1016/j.jacc.2012.11.019

[19] Chevalier P Burri H Fahrat F. Perioperative outcome and long-term survival of surgery for acute post-infarction mitral regurgitation. Eur J Cardiothorac Surg. 2004;26:330–5. 15296892 10.1016/j.ejcts.2004.04.027

[20] Schroeter T Lehmann S Misfeld M. Clinical outcome after mitral valve surgery due to ischemic papillary muscle rupture. Ann Thorac Surg. 2013;95:820–4. 23219255 10.1016/j.athoracsur.2012.10.050

[21] Al-Sarraf N Maher A Muddaiah NK Agzamov Y Jabbour N. The use of veno-veno-arterial ECMO as a successful strategy in acute mitral regurgitation secondary to papillary muscle rupture causing cardiogenic shock and profound hypoxemia: a case report. J Surg Case Rep. 2024;2024:rjae408. 38845794 10.1093/jscr/rjae408 PMC11154825

